# The 2015 global production capacity of seasonal and pandemic influenza vaccine

**DOI:** 10.1016/j.vaccine.2016.08.019

**Published:** 2016-10-26

**Authors:** Kenneth A. McLean, Shoshanna​ Goldin, Claudia Nannei, Erin Sparrow, Guido Torelli

**Affiliations:** a University of Edinburgh, Edinburgh, UK; b Yale School of Public Health, Haven, USA; c World Health Organization, Geneva, Switzerland

**Keywords:** Vaccine, Influenza, Seasonal, Pandemic, Production, Capacity, GAP, Global Action Plan on Influenza Vaccines, EUR, European Region, AMR, Region of the Americas, EMR, Eastern Mediterranean Region, WPR, Western Pacific Region, SEAR, South-East Asia Region, AFR, African Region, LMIC, Low-and middle-income countries

## Abstract

A global shortage and inequitable access to influenza vaccines has been cause for concern for developing countries who face dire consequences in the event of a pandemic. The Global Action Plan for Influenza Vaccines (GAP) was launched in 2006 to increase global capacity for influenza vaccine production to address these concerns. It is widely recognized that well-developed infrastructure to produce seasonal influenza vaccines leads to increased capacity to produce pandemic influenza vaccines. This article summarizes the results of a survey administered to 44 manufacturers to assess their production capacity for seasonal influenza and pandemic influenza vaccine production. When the GAP was launched in 2006, global production capacity for seasonal and pandemic vaccines was estimated to be 500 million and 1.5 billion doses respectively. Since 2006 there has been a significant increase in capacity, with the 2013 survey estimating global capacity at 1.5 billion seasonal and 6.2 billion pandemic doses. Results of the current survey showed that global seasonal influenza vaccine production capacity has decreased since 2013 from 1.504 billion doses to 1.467 billion doses. However, notwithstanding the overall global decrease in seasonal vaccine capacity there were notable positive changes in the distribution of production capacity with increases noted in South East Asia (SEAR) and the Western Pacific (WPR) regions, albeit on a small scale. Despite a decrease in seasonal capacity, there has been a global increase of pandemic influenza vaccine production capacity from 6.2 billion doses in 2013 to 6.4 billion doses in 2015. This growth can be attributed to a shift towards more quadrivalent vaccine production and also to increased use of adjuvants. Pandemic influenza vaccine production capacity is at its highest recorded levels however challenges remain in maintaining this capacity and in ensuring access in the event of a pandemic to underserved regions.

## Highlights

• Significant growth reported in influenza vaccine production capacity since 2006.
• Seasonal influenza vaccine production capacity has reduced since 2013.
• Pandemic influenza vaccine production capacity is at its highest recorded level.
• Challenges remain regarding maintenance of capacity and equitable distribution.

## 1 Introduction

Best estimates indicate that seasonal influenza affects 5–10% of the global population each year, resulting in between 250 and 500 thousand deaths [1]. The case fatality rate (CFR) for pandemic influenza can be substantially greater [2]. In the event of an influenza pandemic, the world will require the rapid production and distribution of billions of doses of vaccine in order to meet expected demand and protect the global population [3].

Global pandemic influenza preparedness is dependent on seasonal influenza vaccine production capacity: in the event of an influenza pandemic, vaccine would be produced in facilities that normally produce seasonal influenza vaccine and the capacity of these facilities defines the global capacity to produce pandemic vaccines. Understanding the operational capacity, trends, and limitations for seasonal vaccine production offers a lens through which the global community can assess its pandemic influenza preparedness.

Launched in 2006, the Global Action Plan on Influenza Vaccines (GAP) was a ten year strategy that aimed to significantly strengthen vaccine production capability for effective response to the pandemic influenza threat [4]. Coordinated by the World Health Organization (WHO), the GAP provided a comprehensive strategy to address global scarcities and inequitable access to influenza vaccines. Within the GAP, global attention was directed towards increasing evidence-based seasonal influenza vaccine use and growing global pandemic vaccine production as well as promoting research and development for improved vaccines. The overall goal of the GAP is to have enough production capacity to immunize the global population within six months of the transfer of the candidate vaccine virus to manufacturers. Taking into account herd immunity and that a single dose may not provide sufficient protection, this means ensuring equitable access to enough vaccine to immunize 70% of the global population with two doses of vaccine [5], [6].

The WHO has continued to perform regular surveys of influenza vaccine manufacturers to compile a projection of global production capacity for both seasonal and pandemic influenza vaccines [6], [7], [8], [9]. This article highlights the international progress and projections of seasonal and pandemic influenza vaccine production capacity through analysis of the 2015 manufacturers’ survey.

## 2 Methods

The WHO surveyed 44 vaccine manufacturers with seasonal influenza vaccine production regarding their seasonal and potential pandemic influenza production capacity for 2015. Vaccine manufacturers that participated in previous WHO production capacity information gathering surveys were asked to confirm or amend information previously collected. Vaccine manufacturers responding for the first time completed a Microsoft Excel® questionnaire, a copy of which is included in Appendix A (see supplementary material). For manufacturers who did not respond to the survey, the WHO incorporated data from previous surveys and publically available information including press releases and public statements. All information provided was triangulated for accuracy.

Each manufacturer was asked to report and/or confirm the estimated maximum total production capacity for 2015 (in millions of doses) of seasonal (Northern and Southern hemisphere) and, if applicable, monovalent pandemic influenza vaccine. Taking this information into consideration, this model forecasted future potential pandemic influenza production capacity. The projection method took into consideration whether the seasonal influenza vaccine was trivalent (with the three predicted strains most likely to circulate): in this case the maximum seasonal production capacity was multiplied by three within the model. In the case of tetravalent seasonal influenza vaccines (with the four predicted strains most likely to circulate) the potential maximum pandemic influenza vaccine production capacity was obtained by multiplying the seasonal capacity by four. For manufacturers with registered pandemic vaccines containing adjuvants the manufacturing capacity was determined by multiplying the seasonal capacity in terms of monovalent doses at 15 μg by the dose-sparing afforded by the adjuvant.

Thirty-five manufacturers (80%) responded to the survey. Manufacturers were approached to clarify when there was a significant conflict among the numbers declared by the manufacturer in terms of production capacity and the previous or publicly available numbers, and the relevant changes were made to the final figures.

Data was aggregated by WHO region to preserve individual manufacturer confidentiality. Furthermore, due to the multinational nature of some of the biggest manufacturers and the way data was reported, the summary information is presented as an aggregated global total.

## 3 Results

### 3.1 Seasonal influenza vaccine production capacity

**Fig. 1:**
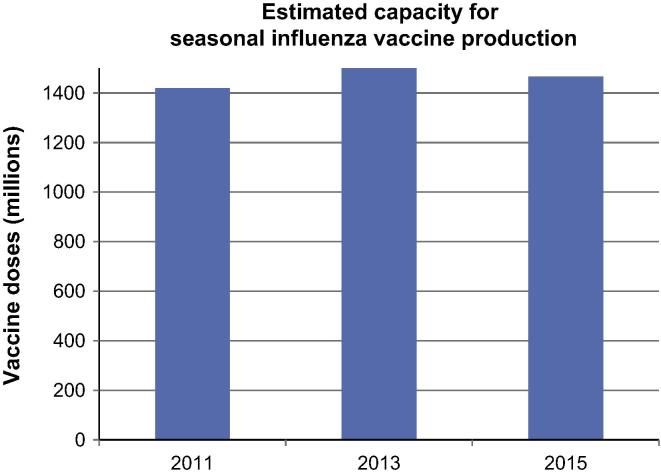
Global seasonal influenza vaccine capacity, year 2011–2015.

In 2015, the global seasonal influenza production is determined to be 1.467 billion doses compared to 1.504 billion doses reported in 2013 [9] and 1.420 billion doses in 2011 [6]. Compared to 2013, this represents a reduction of 37 million doses. This reduction results from several manufacturers either having reduced their capacity or having put their facilities on hold and a subsequent decrease in production at these manufacturers totalling 96 million doses. Fortunately, this is partially offset by a few new manufacturers having established manufacturing processes with a combined capacity of 59 million doses. Fig. 1 details the evolution of global seasonal influenza production capacity from 2011 to 2015.

The majority of production capacity is from manufacturers in high income countries. Thirteen manufacturers are located in lower-middle income countries with a combined capacity of 200 million doses of seasonal influenza vaccine and four manufacturers are in upper-middle income countries with a combined capacity of 250 million seasonal vaccine doses. The remaining 1017 million seasonal vaccine doses are from manufacturers in high income countries.

In the region of the Americas (AMR), one large-scale facility has started production since 2013 resulting in an approximate 40 million increase in seasonal vaccine production capacity in this region. However, one manufacturer reported a reduction in capacity of 60 million doses, and another a reduction of 6 million. These reductions are a function of reduced demand and not an infrastructural change to the facility. However, they do negatively impact the global capacity since these manufacturers have effectively decreased their egg procurement and in the event of a pandemic the egg supply they had previously would not be guaranteed. There is therefore a net decrease in this region of 26 million doses of seasonal vaccine. The impact on pandemic capacity is slightly mitigated by the fact that some of these manufacturers have transitioned from trivalent to tetravalent vaccine. In these cases the same seasonal capacity corresponds to a 33% increase in pandemic capacity as extrapolated from total hemagglutinin production.

In the European region (EUR), over the last three years three small to medium sized manufacturers have put their production on hold, resulting in a reduction of approximately 30 million doses of production capacity. The manufacturers have indicated that this hold is temporary following either refurbishment of their facilities, a change to tissue culture-based technology, or a new market strategy following change in ownership. There is therefore a net decrease of 30 million doses in the region compared to data from previous years. Several of the manufacturers in this region have transitioned from trivalent to tetravalent which means that this small decrease in seasonal capacity does not negatively impact the pandemic capacity.

In South East Asia (SEAR) and the Western Pacific (WPR) regions, five new manufacturers started small-scale production facilities (between 3 and 10 million doses per year per facility) contributing cumulatively a 40 million increase in production capacity since 2013. While these manufacturers have published plans for expansion of their capacities, this study reports what their current feasible capacity is.

Currently, in the African (AFR) and Eastern Mediterranean (EMR) regions there is no active production capacity.

### 3.2 Pandemic influenza vaccine production capacity

In the last four years, the maximum predicted pandemic influenza vaccine production capacity has increased from 4.260 billion doses in 2011 to 6.176 billion doses in 2013 continuing to increase to 6.372 billion doses in 2015.

While the projected global seasonal influenza vaccine capacity has decreased approximately 40 million doses since 2013 (as explained above), the pandemic influenza capacity has risen by approximately 200 million doses.

This pandemic capacity increase despite seasonal capacity decrease is due primarily to multiple manufacturers shifting from trivalent to tetravalent technology. This technology allows more monovalent vaccine doses to be produced within the existing seasonal vaccine production infrastructure.

As in 2013, two large-scale multinational manufacturers, cumulatively producing more than 250 million doses of seasonal influenza vaccine, have approved adjuvanted vaccines that allow antigen dose-sparing within the seasonal influenza vaccine. This technology allows a maximum pandemic production capacity for these two corporations to reach a figure close to 2 billion doses of monovalent pandemic vaccine. Recent mergers in the influenza market suggest that in the near future these dose-sparing adjuvants may be used on a larger scale, however this potential has not been factored into the current estimation.

**Fig. 2:**
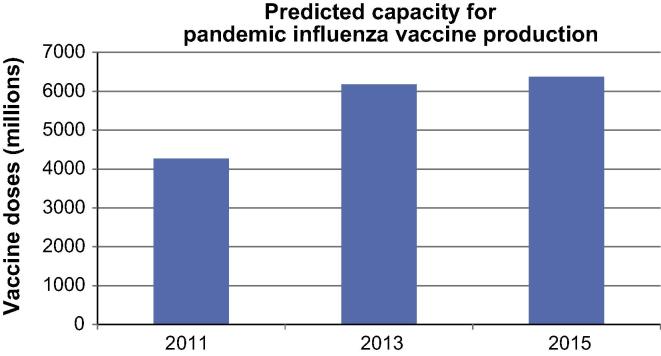
Global pandemic influenza vaccine capacity, year 2011–2015.

Fig. 2 displays projected global pandemic influenza vaccine capacity from 2011 to 2015.

## 4 Discussion

This survey follows on from previous surveys and provides a measure of changes in the global seasonal and pandemic influenza vaccine manufacturing capacity.

When the GAP was initiated in 2006, the estimated vaccine production capacity was just 500 million doses of seasonal vaccine and 1.5 billion doses of pandemic vaccine [4]. From 2006 to 2011 this production capacity grew significantly. Since 2011, a slight growth of seasonal vaccine capacity can be observed, even if slower than in previous years and not evenly geographically spread. This reflects a plateauing of seasonal demand and an off-setting of increased production in some regions by a decreased production in others. The notable decrease of production capacity in the Americas and Europe may indicate a decrease in seasonal demand and uptake in these regions.

Manufacturers in the European region have reported reductions in their 2015 actual production and egg procurement for seasonal influenza vaccine, probably due to a reduced demand within the European market. Within the region of the Americas, two large-scale facilities became operational since 2013, resulting in approximately 90 million additional doses of seasonal influenza vaccine. As these two companies have trivalent and tetravalent technology, they contribute overall to a potential pandemic influenza vaccine production of half a billion doses.

Within SEAR and WPR regions, numerous new small manufacturers have added capacity for approximately 40 million doses per year of seasonal influenza vaccine, and they have announced plans to continue expanding production. The expansion of seasonal influenza vaccine production observed in the WPR and SEAR regions partially offsets the decrease in vaccine production in Europe and the Americas.

Over the last 4 years, the AFR and EMR regions have not developed any functional production capacity for seasonal influenza vaccine, despite some national and sub-regional plans to introduce or increase seasonal vaccine use and progress in acquiring technologies necessary for production.

Over the next five years changes to influenza vaccine production technology are anticipated to impact global production capacity: tissue-culture production, which is not dependent on supply of eggs, may permit greater ramp-up in the event of a pandemic, and in particular production on insect cells has potential for rapid expansion. In addition, several manufactures, including some in developing countries, are establishing adjuvant production and the use of these dose-sparing technologies is anticipated to become more common within the next several years, doubling their current pandemic capacity.

Considering the recent progress in terms of new technologies and new players entering the production field, the predicted global pandemic influenza vaccine production capacity is at its highest. A predicted capacity of 6.372 billion doses, represents enough vaccine to immunize 3.186 billion people with two doses of vaccine in the event of a pandemic, or 43% of the current global population. If one dose of vaccine would offer sufficient protection, as was the case for the 2009 H1N1 pandemic then there would be enough vaccine to cover 86% of the current population.

In order to maintain and expand current production capacity, demand for seasonal influenza vaccines is critical. Ensuring manufacturers with a market for seasonal influenza vaccines will ensure the infrastructure to switch to pandemic vaccine production in the event of an influenza pandemic. The decrease seen in seasonal vaccine production capacity from 2013 to 2015 is of concern. In order to increase seasonal influenza vaccine uptake countries needs to implement policies to vaccinate at-risk groups based on local disease burden, epidemiology of the virus and cost-effectiveness of the vaccine. If influenza immunization rates stagnate or drop this could result in manufacturers reducing or stopping their seasonal vaccine production which will impact the global capacity for pandemic influenza vaccines.

This survey (and the previous) did not evaluate the percentage of prequalified vaccines nor the global capacity of WHO prequalified vaccines. As was seen during the 2009 H1N1 pandemic, the rapid procurement and supply of pandemic vaccines to developing countries is dependent on these vaccines being prequalified. While global capacity increases, and several of the manufacturers have made commitments through the Pandemic Influenza Preparedness (PIP) framework to supply pandemic vaccines to WHO, unless there is an increase in the supply of prequalified pandemic vaccines the response to a pandemic in developing countries will be delayed.

## 5 Study limitations

The interpretation of the data is complex and has changed over time. There were some variations in the interpretation of the questions with some manufacturers assuming an unlimited supply of eggs and reagents when calculating their potential production capacity, and others referring to more realistic production capacity based on what they know to be the egg supply in the event of a pandemic.

This analysis offers a best case scenario projection. Several assumptions that were not considered within this model may impact pandemic influenza preparedness and global capacity. These assumptions are:

(1) The pandemic influenza strain will grow in eggs with the same yield as the seasonal strain. While this was the case with A(H1N1), it was not at all the case with A(H5N1) and in reality the yield may be significantly less than for seasonal vaccine and result in a lower global capacity;
(2) The supply of eggs for vaccine development is not impacted by the pandemic strain. This projection operates under the assumption that the pandemic influenza strain would not impact egg supply. As avian influenza is one of the most probable sources of a pandemic influenza strain, it is possible that egg production would be affected by the virus. In addition, depending on when the pandemic is declared in relation to seasonal vaccine production it is possible that the seasonal egg supply is not immediately available;
(3) Fifteen micrograms of antigen is sufficient for an effective vaccine dose without adjuvant. While fifteen micrograms was a sufficient amount for an effective influenza A(H1N1) vaccine, some A(H5N1) vaccine required ninety micrograms to be effective; and
(4) There would be a sufficient supply of vials, syringes, etc. and functioning transport networks to disperse and dispense the vaccine, and that this would be equitable across populations.

## 6 Conclusion

The estimates in this paper present a best case scenario, one in which all manufacturers would switch to pandemic vaccine production immediately on availability of the candidate vaccine virus and where pandemic vaccine yield and antigen content would be similar to that of seasonal influenza vaccine. Under this scenario, the goal of the GAP to have enough vaccine production capacity to immunize 70% of the global population with two doses of vaccine within six months is not met. If this is spread out over one year and if only one dose of vaccine is needed then the current capacity would be sufficient. Major challenges going forward will be to maintain influenza production capacity at current levels, and to guarantee that all essential downstream processes (e.g. fill-finish operations) are sufficient to meet upstream surges during influenza pandemics. Of concern is the trend in some regions towards a decrease in production capacity. Sustainability and equitable distribution of production capacity remain key concerns, and while there have been recent additions of new manufacturers, some regions remain underserved with little or no production capacity. Furthermore, WHO prequalification of vaccines is crucial in ensuring the delivery of vaccines to developing countries in the event of an influenza pandemic.

## Funding

This work was commissioned and supported by the World Health Organization.

## Conflict of interest statement

No authors have any conflict of interests.
