# Supplementary material for: The 2015 global production capacity of seasonal and pandemic influenza vaccine
Source: Vaccine. 2016 Oct 26;34(45):5410–3. doi: 10.1016/j.vaccine.2016.08.019 (PMC5357707; doi:10.1016/j.vaccine.2016.08.019)
Supplement: Supplementary data 1 [file mmc1.docx]

Supplementary Table: WHO manufacturer survey on the global production capacity of influenza vaccinations.

| **WHO 2015 Survey on the global production capacity of influenza vaccinations** | | **Name:** | **Organisation:** |  |
| --- | --- | --- | --- | --- |
| **Question 1** | - **Did you have a licenced northern hemisphere (NH) seasonal influenza vaccine for the 2015/16 season?** |  |  |  |
| **Question 2** | **Please complete the following information regarding your vaccine(s):** | **NH Vaccine 1** | **NH Vaccine 2** | **NH Vaccine 3** |
|  | - **Registration Date (dd/mm/yy)** |  |  |  |
|  | - **Indication (e.g. age range, population groups)** |  |  |  |
|  | - **What valence is this vaccine?** |  |  |  |
|  | - **Which specific influenza virus strains does this vaccine protect against?** |  |  |  |
|  | - **How many doses of this vaccine are required for vaccine efficacy?** |  |  |  |
|  | - **Does this vaccine contain a live attenuated virus, inactivated viral components, or another source of antigen?** |  |  |  |
|  | - **How many μg of antigen content of each component does the vaccine contain?** |  |  |  |
|  | - **Is this vaccine adjuvanted?** |  |  |  |
|  | - **Is this vaccine egg-based or cell-based?** |  |  |  |
|  | - **In which country(ies) did you produce this NH seasonal vaccine in the 2015/16 season?** |  |  |  |
|  | - **How many doses (in millions) of this NH seasonal vaccine did you produce (release) in the 2015/16 season from each site?** |  |  |  |
|  | - **How many doses (in millions) of this NH seasonal vaccine could you produce working at maximum capacity in the 2015/16 season from each site?** |  |  |  |
| **Question 3** | - **Did you have a licenced southern hemisphere (SH) seasonal influenza vaccine for the 2015 season?** |  |  |  |
| **Question 4** | **Please complete the following information regarding your vaccine(s):** | **SH Vaccine 1** | **SH Vaccine 2** | **SH Vaccine 3** |
|  | - **Registration Date (dd/mm/yy)** |  |  |  |
|  | - **Indication (e.g. age range, population groups)** |  |  |  |
|  | - **What valence is this vaccine?** |  |  |  |
|  | - **Which specific influenza virus strains does this vaccine protect against?** |  |  |  |
|  | - **How many doses of this vaccine are required for vaccine efficacy?** |  |  |  |
|  | - **Does this vaccine contain a live attenuated virus, inactivated viral components, or another source of antigen?** |  |  |  |
|  | - **How many μg of antigen content of each component does the vaccine contain?** |  |  |  |
|  | - **Is this vaccine adjuvanted?** |  |  |  |
|  | - **Is this vaccine egg-based or cell-based?** |  |  |  |
|  | - **In which country(ies) did you produce this SH seasonal vaccine in the 2014/15 season?** |  |  |  |
|  | - **How many doses (in millions) of this SH seasonal vaccine did you produce (release) in the 2015/16 season from each site?** |  |  |  |
|  | - **How many doses (in millions) of this SH seasonal vaccine could you produce working at maximum capacity in the 2015/16 season from each site?** |  |  |  |
| **Question 5** | - **Do you have a licenced monovalent pandemic influenza vaccine?** |  |  |  |
| **Question 6** | **Please complete the following information regarding your monovalent pandemic influenza vaccine(s):** | **Pandemic Vaccine 1** | **Pandemic Vaccine 2** | **Pandemic Vaccine 3** |
|  | - **Registration Date (dd/mm/yy)** |  |  |  |
|  | - **Which specific pandemic influenza virus strain does this vaccine protect against?** |  |  |  |
|  | - **How many doses of this vaccine are required for vaccine efficacy?** |  |  |  |
|  | - **Does this vaccine contain a live attenuated virus, inactivated viral components, or another source of antigen?** |  |  |  |
|  | - **How many μg of antigen content of each component does the vaccine contain?** |  |  |  |
|  | - **Is this vaccine adjuvanted?** |  |  |  |
|  | - **Is this vaccine egg-based or cell-based?** |  |  |  |
|  | - **In which country(ies) did you produce this monovalent pandemic influenza vaccine in the 2015/16 season?** |  |  |  |
|  | - **How many doses (in millions) of this monovalent pandemic influenza vaccine did you produce (release) in the 2015/16 season from each site?** |  |  |  |
|  | - **How many doses (in millions) of this monovalent pandemic influenza vaccine could you produce working at maximum capacity in the 2015/16 season from each site?** |  |  |  |
| **Question 7** | - **Do you have further comments to make with regards to your influenza vaccine production activities? (maximum 200 words).** |  |  |  |
